# Effect of β-Casein Fortification in Milk Protein on Digestion Properties and Release of Bioactive Peptides in a Suckling Rat Pup Model

**DOI:** 10.3390/foods15010026

**Published:** 2025-12-22

**Authors:** Sijia Song, Yingying Lin, Yuning Zhang, Huiyuan Guo

**Affiliations:** 1 Key Laboratory of Functional Dairy, Department of Nutrition and Health, China Agricultural University, Beijing 100191, China; 2 Food Laboratory of Zhongyuan, Luohe 462300, China

**Keywords:** β-casein, milk protein, digestion properties, bioactive peptides, suckling rat pups

## Abstract

β-casein (β-CN) is the predominant casein fraction in breast milk, while current infant milk formula (IMF) contains substantially lower β-CN levels than breast milk. The impact of β-CN fortification on neonatal digestive characteristic and bioactive peptide release remains an understudied area in vivo. This study investigated the effect of β-CN fortification in milk protein on digestion properties and release of bioactive peptides using a suckling rat pup model. Rat pups were, respectively, gavaged with two milk protein solutions: one with ordinary β-CN content (OBCN) and the other with fortified β-CN content (FBCN). The gastric emptying rate, proteolytic efficiency, and peptidomic profiles of intestinal digesta were evaluated. Results indicated that the FBCN group exhibited accelerated gastric emptying into the intestinal phase and enhanced proteolytic efficiency compared to OBCN group. Furthermore, the FBCN group generated greater peptide diversity in the small intestine, with significantly elevated abundance of bioactive peptide candidates exhibiting broader functional spectra. These findings provide additional evidence for the health effects of β-CN fortification in IMF.

## 1. Introduction

Infant milk formula (IMF) based on bovine milk proteins serves as a critical nutritional alternative for infants unable to receive breast milk [1,2]. However, significant compositional differences exist between bovine and breast milk proteins. Bovine milk typically exhibits a whey-to-casein ratio of 20:80, contrasting sharply with the 60:40 ratio in breast milk. Furthermore, β-casein (β-CN) accounts for approximately 65% of total caseins in breast milk versus only 40% in bovine milk [3,4,5]. The modern benchmark for assessing protein quality, the Digestible Indispensable Amino Acid Score (DIAAS), reveals that bovine casein consistently demonstrates higher values than whey protein, such as a DIAAS of 126 for casein compared to 96 for whey for infants [6,7]. This underscores that casein is not only a structural protein but also an excellent source of highly bioavailable essential amino acids. Currently, the vast majority of IMFs have achieved a whey protein to casein ratio of 60:40 by adding whey powder. However, comparatively less attention has been paid to compositional differences in casein fractions between bovine milk and breast milk in IMF manufacture, especially β-CN. While whey proteins dominate the protein fraction in breast milk and are renowned for their rapid digestibility and rich content of essential amino acids and immunomodulatory components (e.g., lactoferrin), β-CN plays a structurally and functionally distinct role. Its loose, open conformation and high density of phosphorylated serine residues make it the primary carrier of calcium [1,8]. More importantly, β-CN is considered the principal precursor of a wide range of bioactive peptides released during digestion [9]. As the predominant casein in human milk, enhancing β-CN content in IMFs could narrow this compositional gap with breast milk [9,10].

In recent years, an increasing number of studies have shown that the composition of milk proteins has a significant impact on digestion properties [9,11]. Since the first IMF based on whey protein rather than casein was introduced in 1961, considerable attention has focused on how protein composition influence digestive characteristics—particularly in infants [9]. Some researchers have reported that bovine milk’s native 80:20 casein-to-whey ratio forms firmer gastric curds in infants, potentially causing digestive discomfort, whereas adjusting this ratio to 40:60 mitigates such effects [12,13]. Subsequent studies have suggested further adjustments to the subcomponents of casein, specifically increasing the proportion of β-CN in casein from 40% to around 65%, which can make the digestion properties of milk protein more similar to that of human milk [14]. Huang et al. used an in vitro infant digestion model and found that the β-CN-fortified formulas exhibited higher protein hydrolysis degree, elevated free amino acid content, and improved essential amino acid bioavailability compared to controls, achieving digestive outcomes closer to human milk [9]. Critically, β-CN-derived peptides predominated across all formulas, confirming β-CN’s role as a primary precursor of bioactive peptides in milk proteins. While numerous studies have investigated β-CN fortification effects on infant digestion, most rely on in vitro models. Studies using animal models, especially suckling offspring, are still relatively few and require further exploration.

The Sprague–Dawley (SD) rat is one of the most commonly used experimental models for in vivo digestion studies. Its docile temperament, larger body size compared to mice (allowing greater sample intake to reduce experimental error), cost-effectiveness, and widespread availability make it suitable for food digestion research in most laboratories. Consequently, SD rats represent an optimal model for investigating food nutritional properties. There have already been numerous reports using the SD rat model to investigate the in vivo digestion characteristics of milk proteins [15,16,17]. Wada et al. used the SD rat pup model to analyze peptides released from milk proteins in human milk and IMF, and found that α-lactalbumin and β-CN in human milk, as well as β-lactoglobulin and β-CN in IMF, are the main sources of peptides [15].

Therefore, the objective of this study was to investigate the effect of β-CN fortification in milk protein on digestion properties and release of bioactive peptides using a suckling rat pup model. Firstly, digestion rate of the proteins was detected by fluorescence labeling and tracking method. Then, the proteolytic efficiency was analyzed through sodium dodecyl sulfate-polyacrylamide gel electrophoresis (SDS-PAGE). Finally, the peptidomic profiles of intestinal digesta was characterized using nanoLC-MS/MS. These data might provide additional evidence for the health effects of β-CN fortification in IMF.

## 2. Materials and Methods

### 2.1. Materials

Bovine β-CN (83% purity, lots SLCB7950) and protease inhibitor cocktail were purchased from Sigma Aldrich (St. Louis, MO, USA). Casein (92% purity) was purchased from Shanghai Yuanye Bio-Technology Co., Ltd. (Shanghai, China). Whey protein isolate (90% purity) was purchased from Hilmar Ingredients (Hilmar, CA, USA). Sulfo-cyanine7 succinimidyl ester (CY7-SE) was purchased from MedChemExpress (Elizabeth, NJ, USA). The other chemical reagents used were all analytical grade.

### 2.2. In Vivo Digestion

#### 2.2.1. Preparation of Milk Protein Solution for Gavage

Two types of gavage solutions were prepared: a milk protein with ordinary β-CN proportion (OBCN, the proportion of β-CN in casein is 43.0%) and a milk protein with fortified β-CN proportion (FBCN, the proportion of β-CN in casein is 63.4%). The ingredients used in two types of solutions are shown in Table 1. The total protein concentration of the gavage solutions was 20 mg/mL, with a casein to whey protein ratio of 40:60 in both solutions. The protein was dissolved in phosphate-buffered saline (PBS) and stirred overnight at 4 °C.

#### 2.2.2. Grouping and Treatment of Experimental Animals

A suckling rat pup model was used to study in vivo digestion of the milk protein samples according to the method of Wada et al. with slight modifications [15]. Animal experiments were conducted with the approval of the Laboratory Animal Welfare and Animal Experimental Ethics Review Committee of China Agricultural University (Aw81104202-4-2). Sprague–Dawley (SD) rat pups were obtained commercially (Charles River, Beijing, China). After acclimation to facility conditions, pups were separated from their dam for 12 h on postnatal day 14. Thirty-six pups were randomly divided into two groups and gavaged with 1 mL OBCN and FBCN solutions, respectively. The pups were sacrificed at 30, 60, and 120 min after gavage, with six rats sacrificed at each time point. The contents of the stomach, upper small intestine, and lower small intestine were collected. The stomach contents were weighed immediately and then quickly transferred to a sterile centrifuge tube containing 0.1 mL of protease inhibitor cocktail solution. The small intestine was divided into upper and lower parts, each of which was perfused with 0.4 mL of protease inhibitor cocktail solution and collected into a sterile centrifuge tube. The collected gastrointestinal digesta samples were rapidly frozen in liquid nitrogen and stored at −80 °C until analysis.

### 2.3. Protein Digestion Rate in the Gastrointestinal Tract

The protein digestion rate in the gastrointestinal tract was detected by fluorescence labeling and tracking method following the method of Li et al. with slight modifications [18]. Nine 14-day-old rat pups were separated from their dam for 12 h and then randomly divided into three groups, with three pups in each group. OBCN and FBCN solutions (10 mg/mL) were labeled with CY7-SE according to the manufacturer’s instructions. Each pup in the experimental groups was gavaged with 1 mL Cy7-labeled OBCN or FBCN solutions, while each pup in the control group was gavaged with 1 mL PBS. At 30 min after gavage, the pups were sacrificed, and their gastrointestinal tracts were dissected and placed in 90 mm plates. Fluorescence signals were observed and photographed using the In Vivo Imaging System (IVIS) Spectrum (Perkin Elmer, Waltham, MA, USA). The emission wavelength and excitation wavelength of CY7-SE are 710 nm and 760 nm, respectively.

### 2.4. SDS-PAGE Analysis of Gastrointestinal Digesta

The total protein content of gastrointestinal digesta was determined by the BCA method according to the manufacturer‘s instructions and the protein composition was analyzed by SDS-PAGE [2]. A 50 µL aliquot of gastrointestinal digesta was mixed with 50 µL of sample buffer and boiled for 5 min. A 12% separating gel and a 6% stacking gel were prepared for electrophoresis, with a loading amount of 30 µg for each sample. After electrophoresis, the gels were stained with Coomassie Brilliant Blue R250 for 30 min, followed by destaining.

### 2.5. Peptidomic Analysis

The contents of the upper small intestine of rat pups digested for 30 min were detected by nanoLC-MS/MS for peptidomics analysis [15]. After lyophilization, re-dissolution, desalting, and concentration, the samples were re-dissolved in mobile phase A for injection. Approximately 200 ng of total peptides from each sample were separated by nano-UPLC and then subjected to data acquisition by the mass spectrometer. A PePSep C18 reversed-phase chromatography column was used. Mobile phase A was a 0.1% formic acid aqueous solution, and mobile phase B was a 0.1% formic acid acetonitrile solution. DDA data acquisition was performed in DDA PaSEF mode, with an MS scan range of 100–1700 m/z. The composition and characteristics of the overall identified peptides were analyzed. Venn diagrams were used to characterize the differences in peptide numbers between OBCN and FBCN groups. Peptides with significant differences between groups were further screened and visualized using heatmaps and volcano plots. The sequences of peptides with significant difference were compared with the previously reported bioactive peptides in the Milk Bioactive Peptide Database (MBPDB, http://mbpdb.nws.oregonstate.edu/ (accessed on 14 March 2024)) [19]. Peptide fingerprinting analysis of β-CN-derived peptides from OBCN and FBCN groups was performed using Peptigram platform (http://bioware.ucd.ie/peptigram/ (accessed on 11 April 2024)) [20].

### 2.6. Statistical Analysis

All the experiments were performed in triplicate. The data were analyzed with SPSS version 23.0 (SPSS Inc., Chicago, IL, USA). The normality of data distribution was assessed using the Shapiro–Wilk test. All datasets were consistent with a normal distribution (*p* > 0.05). Therefore, differences between the two groups (OBCN vs. FBCN) were analyzed using an unpaired two-tailed Student’s *t*-test. Differences at *p* < 0.05 were considered as significant.

## 3. Results and Discussion

### 3.1. Digestion Rate of Milk Protein in the Gastrointestinal Tract of Suckling Rat Pups

**Figure 1:**
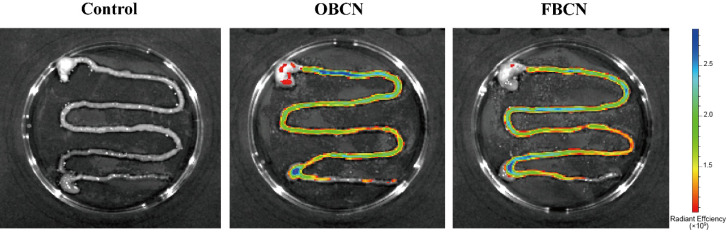
Digestion rate of milk protein in the gastrointestinal tract of suckling rat pups. OBCN: milk protein with ordinary β-casein ratio; FBCN: milk protein with fortified β-casein ratio.

Fluorescence signals of Cy7-labeled OBCN and FBCN were observed and photographed, as shown in Figure 1. The color in the figure indicates the intensity of fluorescence, with blue representing higher fluorescence intensity and thus a greater amount of residual protein. After 30 min of gavage, there was still a large amount of red fluorescence signal in the stomach of the OBCN group, with strong blue fluorescence signals in the upper part of the small intestine, indicating that a significant amount of protein remained in the stomach and upper part of the small intestine of the rat pups. In contrast, the FBCN group had fewer fluorescence signals in the stomach and stronger fluorescence signals in the middle and lower parts of the intestine, indicating that only a small amount of protein remained in the stomach, with a large amount of protein having moved into the middle and lower intestinal segments. These results suggest that, compared to OBCN sample, FBCN sample is more easily emptied from the stomach into the intestinal digestion phase, which may be more digestive-friendly for infants. This finding is consistent with previous studies, where some researchers have found that increasing the proportion of β-CN in milk protein to levels closer to that of human milk can improve the digestion rate of the protein, giving it a digestion pattern similar to that of human milk protein [9,21,22]. Whereas prior research has largely relied on in vitro hydrolysis kinetics to infer digestive rates, our study extends beyond this approach by providing a direct visualization of gastric emptying dynamics in a physiologically relevant suckling pup model [9,11]. Notably, the gastric emptying rate of fluorescence-labeled protein was significantly accelerated in the FBCN group, thus offering novel visual evidence that corroborates the superior digestion property demonstrated in previous in vitro studies.

### 3.2. In Vivo Digestion of Milk Protein Samples

#### 3.2.1. The Weight of Milk Protein Curds in the Stomach of Suckling Rat Pups

**Figure 2:**
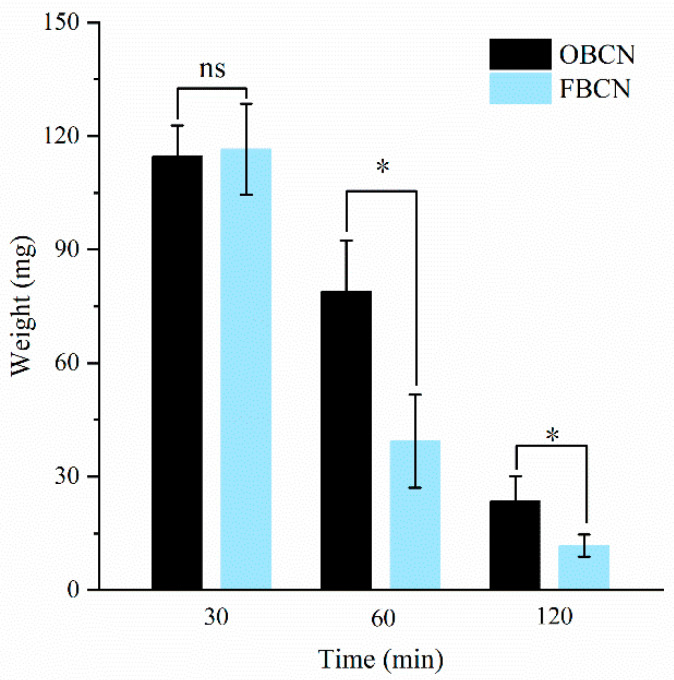
The weight of milk protein curds in the stomach of suckling rat pups. OBCN: milk protein with ordinary β-casein ratio; FBCN: milk protein with fortified β-casein ratio. * *p* < 0.05, ns indicated no significant difference (*p* > 0.05).

The isoelectric point of casein is at pH 4.6. When milk protein enters the stomach of rat pups and comes into contact with gastric juice, the pH gradually decreases to near the isoelectric point, forming solid or semi-solid curds. The size and structure of these curds directly affect the retention time of milk proteins in the stomach, thereby influencing the digestion rate and bioavailability of milk proteins [23,24,25]. The changes in the weight of gastric curds formed by OBCN and FBCN during digestion are shown in Figure 2. The weight of gastric curds both decreased with the extension of digestion time, indicating that the protein were gradually digested and decomposed in the stomach. At 30 min of digestion, there was no significant difference in the weight of gastric curds between the OBCN and FBCN groups (*p* > 0.05). At 60 and 120 min post-digestion, the gastric curd weight in the FBCN group (39.4 ± 12.3 mg and 11.8 ± 2.9 mg, respectively) was approximately 50% lower than that in the OBCN group (78.9 ± 13.5 mg and 23.5 ± 6.7 mg, respectively; both *p* < 0.05), indicating a faster digestion rate of FBCN curds. It is speculated that the increased β-CN content in FBCN makes it more easily digested in the stomach of rat pups. This result is consistent with previous research, where Huang et al. conducted in vitro infant digestion on milk protein formulas containing different levels of β-CN and observed that the groups with appropriate β-CN fortification exhibited faster protein digestion in gastric fluid [9].

#### 3.2.2. SDS-PAGE of Milk Protein in the Gastrointestinal Digesta of Suckling Rat Pups

**Figure 3:**
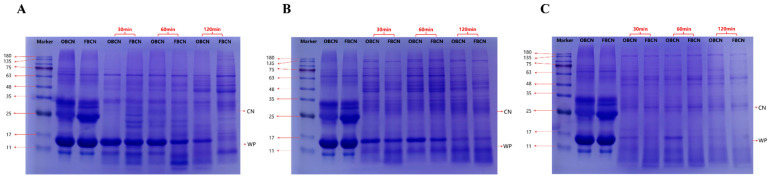
SDS-PAGE of milk protein in the gastric (**A**), upper (**B**) and lower (**C**) small intestinal digesta of suckling rat pups. OBCN: milk protein with ordinary β-casein ratio; FBCN: milk protein with fortified β-casein ratio.

The protein composition of the digesta after OBCN and FBCN digestion for 30, 60, and 120 min was analyzed, as shown in Figure 3. Figure 3A shows the changes in protein composition in the stomach contents over time. The first three lanes correspond to the protein marker, the prepared OBCN solution, and the FBCN solution, respectively. With the increase in digestion time, the overall protein bands in both groups became progressively lighter, indicating that the proteins in the stomach were gradually emptied into the intestines. At 60 min of digestion, the protein bands in the FBCN group were already significantly lighter than those in the OBCN group, indicating that less protein remained in the stomach of the FBCN group. This difference became even more pronounced at 120 min of digestion, suggesting that the FBCN was more readily digested and emptied from the stomach. It is speculated that the increased β-CN content in the FBCN makes it more easily digested in the stomach of rat pups. Figure 3B,C show the changes in protein composition in the contents of the upper and lower parts of the small intestine, respectively. As time progressed, the overall protein bands in both groups became progressively lighter. The protein bands in the FBCN group were slightly lighter than those in the OBCN group, indicating that the FBCN seemed to be more easily digested and absorbed in the intestines than the OBCN.

The accelerated gastric emptying and enhanced proteolytic efficiency observed in the FBCN group can be linked to the distinct physicochemical properties of β-CN. Compared to other caseins, β-CN possesses a more open and flexible structure with a unique distribution of phosphorylated serine residues. This structural characteristic likely contributes to the formation of looser, more porous curds upon gastric acidification [3,9]. Such a porous curd structure offers a larger surface area for pepsin penetration and proteolytic attack, facilitating faster protein breakdown. Consequently, the rapid hydrolysis reduces the curd’s cohesiveness and viscosity, promoting its disintegration and faster transit from the stomach.

### 3.3. Peptidomic Analysis of Intestinal Digesta of Suckling Rat Pups

Peptidomic analysis of intestinal digesta from rat pups fed OBCN versus FBCN was conducted and a total of 1906 unique peptides were identified across both groups. The composition and characteristics of the overall peptides were analyzed.

#### 3.3.1. Analysis of Differential Peptide Originated from Milk Protein

**Figure 4:**
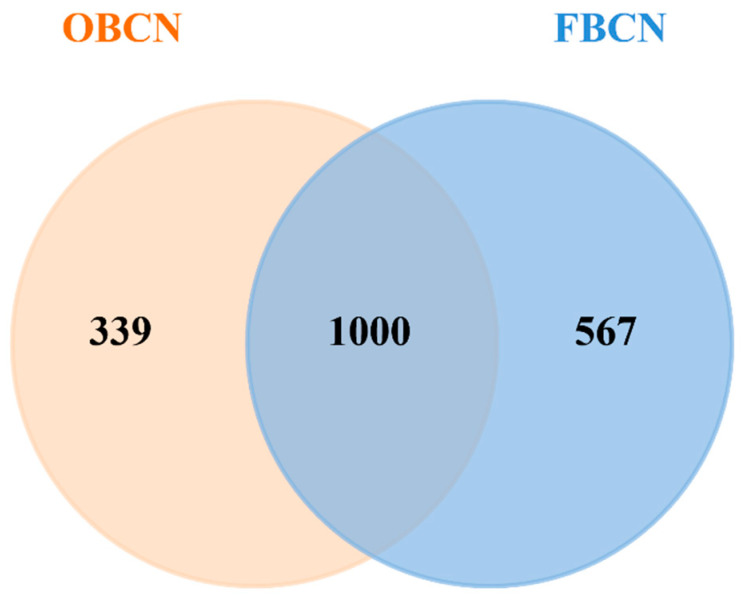
Number of peptide species originated from milk protein in the small intestine of suckling rat pups. OBCN: milk protein with ordinary β-casein ratio; FBCN: milk protein with fortified β-casein ratio.

The overlap and differences in peptide species between OBCN and FBCN groups were visually presented in Figure 4. Digestion generated 1339 and 1567 distinct peptides in the OBCN and FBCN groups, respectively, with 1000 shared peptides (see Supplementary Table S1). Notably, the FBCN digesta contained 567 group-specific peptides, which was 67.3% more than the 339 found uniquely in the OBCN digesta. This indicates that the digestion of FBCN in the gastrointestinal tract of rat pups produced a greater variety of peptides than OBCN. This may be due to the enrichment of β-CN in the sample, as β-CN is one of the main sources of milk protein peptides, resulting in a larger variety of peptides produced in the FBCN group [9,15].

**Figure 5:**
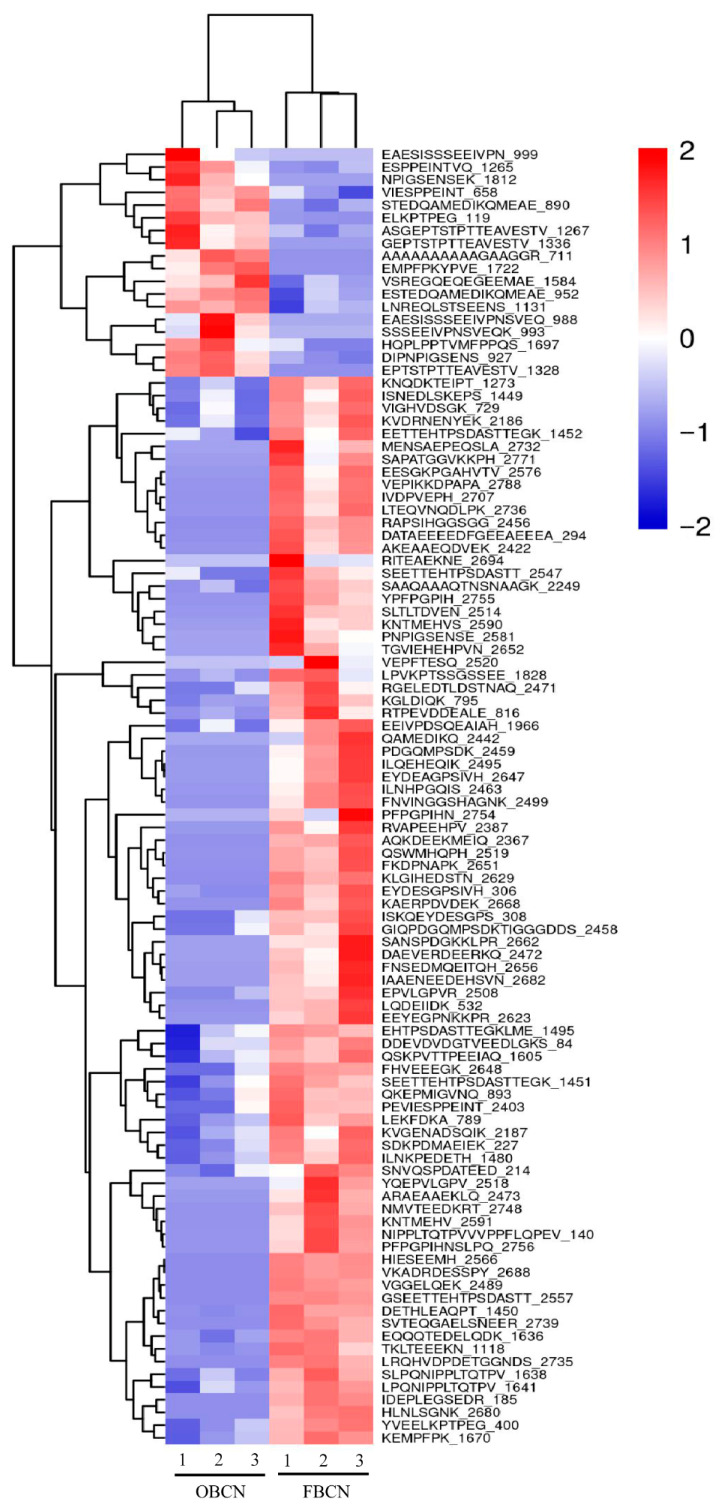
Cluster analysis of the differential peptides originated from milk protein in the small intestine of suckling rat pups. OBCN: milk protein with ordinary β-casein ratio; FBCN: milk protein with fortified β-casein ratio.

Differentially abundant peptides between OBCN and FBCN groups were screened and subjected to hierarchical clustering analysis, with the results presented in a heatmap. The heatmap intuitively reflects the similarities and differences between samples through color variations. As shown in Figure 5, the three samples of OBCN clustered together, while FBCN samples formed a distinct cluster, indicating minimal intra-group variations. However, there were substantial differences in the color blocks between OBCN and FBCN groups, with the vast majority of peptides exhibiting elevated abundance in FBCN group. These patterns demonstrate significant enhancement in peptide yield from FBCN during digestion compared to OBCN.

**Figure 6:**
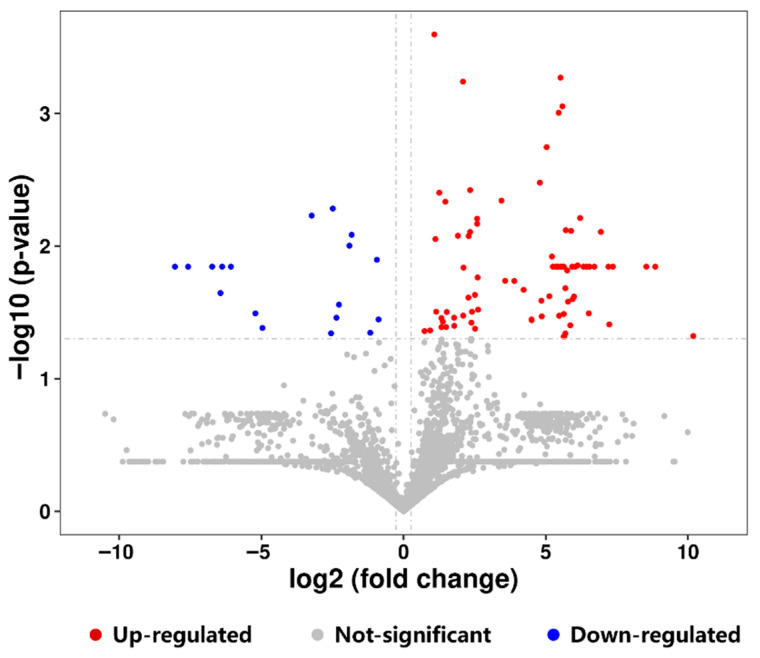
Volcano plot of the differential peptides originated from milk protein in the small intestine of suckling rat pups.

The volcano plot intuitively displays the significant changes in peptides between the two groups of samples, as shown in Figure 6. Each point in the figure represents a detected peptide, with the *x*-axis indicating the fold change in each peptide. Significantly upregulated peptides are shown in red, while significantly downregulated peptides are shown in blue. Comparative analysis revealed 84 significantly upregulated peptides and 18 significantly downregulated peptides in the FBCN group versus the OBCN group. Among the significantly upregulated peptides, those derived from β-CN were the most numerous, with 13 peptides. The detailed peptide information was presented in Supplementary Table S2.

#### 3.3.2. Analysis of Potentially Bioactive Peptides Originated from Milk Protein

The differentially abundant peptides (84 upregulated, 18 downregulated) between OBCN and FBCN groups were aligned against bioactive peptides in the MBPDB to compare bioactive peptide generation. Alignment parameters included 80% sequence homology with allowance for conservative substitutions (based on similar physicochemical properties, specific amino acids were considered equivalent substitutions, such as valine, isoleucine, and leucine; aspartic acid and glutamic acid; arginine and lysine) to identify bioactive candidates [19]. The results are shown in Table 2.

Among the significantly upregulated peptides in the FBCN group, 10 exhibited bioactive potential with functions including: antimicrobial activity, immunomodulatory, antioxidant activity, angiotensin I-converting enzyme (ACE) inhibitory activity, increased mucin secretion, opioid, antianxiety, dipeptidyl peptidase (DPP) IV inhibitory activity, and antithrombotic activity. As for the significantly downregulated peptides, 4 exhibited bioactive potential, involving immunomodulatory, antioxidant activity, and ACE-inhibitory activity. In addition, among 10 significantly upregulated potential bioactive peptides, 8 are derived from β-CN. The peptides RTPEVDDEALE, KEMPFPK and PFPGPIHNSLPQ has the potential for antimicrobial activity, and may protect infants from bacterial infections. The peptides EPVLGPVR, YPFPGPIH and YQEPVLGPV has the potential for immunomodulatory activity, and may help infants maintain immune balance. The peptides RTPEVDDEALE, EPVLGPVR, YPFPGPIH, YQEPVLGPV and YVEELKPTPEG has the potential for antioxidant activity, and may protect infants from oxidative stress. The quantitative analysis revealed a substantial enrichment of antimicrobial peptides in the FBCN group, with the total relative abundance of identified sequences such as RTPEVDDEALE, KEMPFPK, and PFPGPIHNSLPQ being 6.4-fold greater than in the OBCN group. Notably, the peptide KEMPFPK exhibited the highest individual abundance. This finding gains functional relevance when considering its closely homologous sequences reported in the literature; the peptide EMPFPK demonstrates relatively low minimum inhibitory concentrations (MICs) against *Staphylococcus aureus* (20 µg/mL) and *Escherichia coli* (60 µg/mL), while HKEMPFPK shows MICs of 25 µg/mL and 50 µg/mL against the same pathogens, respectively. The combination of their low reported MICs and the significantly elevated abundance of KEMPFPK in our in vivo digest suggests that β-CN fortification may not only increase the output of antimicrobial peptides but also enrich peptides whose structural homologs are reported to be active at low concentrations, thereby potentially contributing to a fortified gastrointestinal defense. These results show that the FBCN group enhances abundance of bioactive peptides generated during gastrointestinal digestion in rat pups, suggesting that fortification of β-CN in milk protein may provide more bioactive peptides for the protection of infants.

#### 3.3.3. Peptide Profile Originated from β-CN

**Figure 7:**
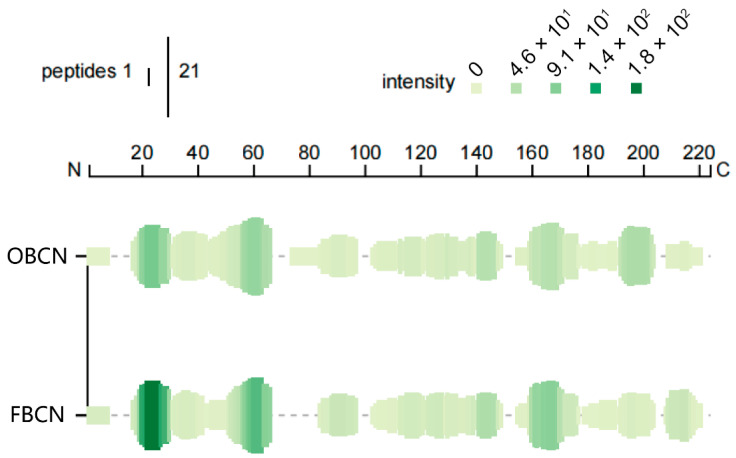
Peptide profile originated from β-casein in the small intestine of suckling rat pups. OBCN: milk protein with ordinary β-casein ratio; FBCN: milk protein with fortified β-casein ratio.

Peptide profile involves matching the peptide sequences with the amino acid sequence of the original protein, thereby globally displaying the peptide diversity, cleavage sites, and abundance [57]. Peptides derived from β-CN in OBCN and FBCN groups were selected to construct peptide profile, which intuitively show the differences in β-CN degradation patterns, as shown in Figure 7. Each group occupies a dedicated row, with vertical green bars indicating amino acid positions covered by at least one peptide. Bar height corresponds to the number of distinct peptides at each position, while color intensity reflects cumulative peptide abundance, with dark green representing a high relative abundance. The figure shows that peptides originated from β-CN in both groups almost cover the entire β-CN sequence, indicating that β-CN can be fully digested in the gastrointestinal tract of rat pups. This is consistent with previous studies, where researchers reported that the secondary structure of β-CN is relatively loose and the monomeric conformation is open, making it easily digestible in the infant gut [58]. Moreover, the diversity and abundance of peptides originated from β-CN in OBCN group are much higher than those in FBCN group, indicating that β-CN fortification can promote the release of more peptides in the intestines of rat pups, providing a foundation for its biological functions.

The distinct effects of β-CN fortification observed in our study can be attributed to its unique physicochemical properties among casein variants. Structurally, β-CN is characterized by a high proportion of proline residues, which disrupt regular secondary structures, resulting in a more open and flexible conformation compared to the more compact and highly phosphorylated αs1-casein [3]. This “looser” structure, coupled with its strong amphiphilicity (a clearly separated hydrophilic N-terminal and hydrophobic C-terminal region), governs its behavior during digestion. In the gastric environment, β-CN forms softer, more porous curds due to weaker intermolecular interactions, which significantly increases the surface area accessible to proteases like pepsin [9], thereby enhancing proteolytic efficiency and accelerating gastric emptying—a key finding of our in vivo model. Furthermore, its amino acid sequence is enriched in specific cleavage sites for digestive enzymes and serves as a primary precursor for a wide array of bioactive peptides [3,11]. Therefore, increasing its concentration not only improves the physical dynamics of digestion but also directly amplifies the pool of peptide sequences with high potential for biological activity, explaining the more diverse and abundant bioactive peptide profile identified in the FBCN group.

This study provides the first comprehensive in vivo evidence, obtained using a physiologically relevant suckling rat pup model, to systematically evaluate the impact of β-CN fortification, thereby offering direct physiological validation beyond the limitations of prior in vitro analyses. It demonstrates that β-CN fortification actively impacts the digestive process, which accelerates gastric emptying and contributes to a more diverse bioactive peptide profile. While previous research has identified β-CN as a peptide precursor in vitro [9], our work provides direct in vivo evidence that increasing its concentration actively shapes a more diverse and functionally expansive peptide landscape. These findings advance β-CN fortification from a general nutritional adjustment to a targeted strategy for optimizing digestive outcomes. However, certain limitations must be considered when interpreting these results. While the peptidomic analysis confirms the enriched presence of bioactive peptides (e.g., antimicrobial, immunomodulatory) in the intestinal digesta, their bioavailability, absorption, and consequent in vivo physiological efficacy remain to be validated. Furthermore, the discussion of their functional potential is inferred from potency data reported in in vitro assays, and extrapolating these effects to the complex in vivo milieu involves inherent constraints. Finally, while the sample size in this exploratory study was sufficient to detect substantial effects for the primary endpoints, future confirmatory studies with larger cohorts are warranted. Subsequent research should aim to directly measure the absorption of key peptides and their specific bioactivities in vivo to solidify these conclusions and fully explore their implications for infant nutrition and health.

## 4. Conclusions

In this study, an in vivo digestion model of suckling rat pups was employed to evaluate the digestion properties of β-CN-fortified milk protein, including gastric emptying rate, proteolytic efficiency, and peptidomic profiles of intestinal digesta. The results indicated that the β-CN-fortified milk protein exhibited accelerated gastric emptying into the intestinal phase and enhanced proteolytic efficiency compared to ordinary milk protein. Furthermore, β-CN fortification generated greater peptide diversity in the small intestine, with significantly elevated abundance of bioactive peptide candidates exhibiting broader functional spectra. These findings collectively reveal that β-CN fortification can modify the gastrointestinal digestive fate of milk protein, leading to a peptide profile with potentially broader biofunctional spectra. This provides a novel theoretical basis for the targeted application of β-CN in IMF to mimic potentially beneficial digestive outcomes.

## Figures and Tables

**Table 1:** Ingredients used for preparation of milk protein solutions with different contents of β-casein.

Ingredients (mg)	OBCN	FBCN
Whey protein isolate	266.9	267.8
Casein	174.0	94.7
β-CN	-	89.6
Whey protein/casein ratio	60:40	60:40
Total protein content (mg/mL)	20.0	20.1
β-casein content in casein (%)	43.0%	63.4%

OBCN: milk protein with ordinary β-casein ratio; FBCN: milk protein with fortified β-casein ratio.

**Table 2:** Database-driven prediction of bioactive peptides originated from milk protein in the small intestine of suckling rat pups.

Classification	Peptide Sequence ^a^	Bioactive Sequence (MBPDB) ^b^	Bioactivity ^c^	Parent Protein	References ^d^
Significantly upregulated	RTPEVDDEALE	TPEVDDEALEK, TPEVDKEALE, TPEVDKEALEK	Antimicrobial, Antioxidant, DPP-IV Inhibitory	β-LG	[26,27,28,29,30]
	KEMPFPK	EMPFPK, HKEMPFPK	Antimicrobial, ACE-inhibitory, Increase mucin secretion	β-CN	[31,32,33,34]
	EPVLGPVR	EPVLGPVRGP, YQEPVLGPVR	Immunomodulatory, Antioxidant, ACE-inhibitory, Antithrombotic	β-CN	[35,36,37,38]
	YPFPGPIH	VYPFPGPI, YPFPGPIP, YPFPGPI	Immunomodulatory, Increase mucin secretion, Opioid, ACE-inhibitory, Antianxiety, Antioxidant	β-CN	[39,40,41,42,43,44,45,46,47]
	PFPGPIHNSLPQ	PFTGPIPNSLPQ	Antimicrobial	β-CN	[29]
	YQEPVLGPV	YQEPVLGP, YQEPVLGPVRG, YQEPVLGPVR, LYQEPVLGPVR	Immunomodulatory, Antioxidant, ACE-inhibitory	β-CN	[35,36,48,49,50]
	YVEELKPTPEG	YVEELKPTPEGDL	Antioxidant	β-CN	[51]
	NIPPLTQTPVVVPPFLQPEV	NIPPLTQTPVVVPPFLQ	ACE-inhibitory	β-CN	[52]
	KGLDIQK	GLDIQK	ACE-inhibitory, Cholesterol regulation	β-LG	[31,53]
	PFPGPIHN	PFPGPIPN	ACE-inhibitory	β-CN	[54]
Significantly down-regulated	DIPNPIGSENS	FSDIPNPIGSE, FSDIPNPIGSEN	Antioxidant	α_s1_-CN	[55]
	HQPLPPTVMFPPQS	HQPHQPLPPTVMFPPQ	Immunomodulatory, ACE-inhibitory	β-CN	[50]
	EMPFPKYPVE	MPFPKYPVEP	ACE-inhibitory	β-CN	[52]
	EAESISSSEEIVPNSVEQ	QMEAESISSSEEIVPNSVEQK	Immunomodulatory	α_s1_-CN	[56]

^a^ Peptide sequences were greater than 80% match to reported bioactive peptide [15]. ^b^ Sequences for bioactive peptides were matched to a milk bioactive peptide database [15]. ^c^ DPP-IV inhibitory: dipeptidyl peptidase-IV inhibitory activity, ACE-inhibitory: Angiotensin I-converting enzyme inhibitory activity. ^d^ References are listed in Supplementary Table S3.

## Data Availability

The original contributions presented in the study are included in the article/Supplementary Materials. Further inquiries can be directed to the corresponding author.

## Supplementary Materials

The following supporting information can be downloaded at: https://www.mdpi.com/article/10.3390/foods15010026/s1. Table S1: List of identified peptide segments; Table S2: List of significantly upregulated and downregulated peptides.
